# Glandular Epithelium as a Possible Source of a Fertility Signal in *Ectatomma tuberculatum* (Hymenoptera: Formicidae) Queens

**DOI:** 10.1371/journal.pone.0010219

**Published:** 2010-04-19

**Authors:** Riviane Rodigues da Hora, Jacques Hubert Charles Delabie, Carolina Gonçalves dos Santos, José Eduardo Serrão

**Affiliations:** 1 Departamento de Biologia Geral, Universidade Federal de Viçosa, Viçosa, Brazil; 2 UPA Laboratório de Mirmecologia, Convênio UESC-CEPEC/CEPLAC, Itabuna, Brazil; Institute of Evolutionary Biology (CSIC-UPF), Spain

## Abstract

The wax layer covering the insect's cuticle plays an important protective role, as for example, uncontrolled water loss. In social insects, wax production is well-known in some bees that use it for nest building. Curiously, mated-fertile queens of the ant *Ectatomma tuberculatum* produce an uncommon extra-wax coat and, consequently queens (mated-fertile females) are matte due to such extra cuticular hydrocarbon (CHC) coat that covers the cuticle and masks the brightness of the queens' cuticle while gynes (virgin-infertile queens) are shiny. In this study, histological analysis showed differences in the epidermis between fertile (i.e., queens or gynes with highly ovarian activity) and infertile females (gynes or workers with non developed ovaries). In fertile females the epidermis is a single layer of cubic cells found in all body segments whereas in infertile females it is a thin layer of flattened cells. Ultrastructural features showed active secretory tissue from fertile females similar to the glandular epithelium of wax-producing bees (type I gland). Different hypotheses related to the functions of the glandular epithelium exclusive to the *E. tuberculatum* fertile queens are discussed.

## Introduction

The cuticle of insects is characterized by its rigidity and its multifunctional role includes protection against natural enemies and environmental constraints [e.g. 1], [2]. The outer surface of an insect's cuticle is a thin wax layer that plays an important protective role [3].

The presence of a large amount of wax on the cuticle surface is common in scale insects (Hemiptera) and provides mechanical protection and defense [4], [5]. In some coccinellid (Coleoptera) and sawfly (Hymenoptera) larvae white tufts of wax render the insect unpleasant and deter attacks by natural enemies [6]–[10]. In social insects, wax production is well-known in honey bees, bumble bees and stingless bees that use it for nest building [11]. Esters are the major constituents of wax that are released by bees [12], which are released by epidermal glands [13]–[15].

Lipids of the epicuticle also provide an important waterproofing barrier and several insects produce wax esters and other lipids like hydrocarbons that can reduce excessive water loss through the cuticle [16]–[20]. This waterproofing function of wax was nicely demonstrated in the desert tenebrionid beetle *Cryptoglossa verrucosa*, which produces higher hydrophobic wax cover at low humidity, specifically reducing water loss in this condition [21]. In addition to the cuticular protective role, long-chain cuticular hydrocarbons (CHC) have been largely demonstrated to serve as nestmate and caste recognition and fertility cues in social insects [reviews: 22], [23].

An intriguing finding was recently described in the ant *Ectatomma tuberculatum* (Ectatomminae), where mated-fertile queens produce a wax coat contrarily to unmated infertile ones (gynes). Such wax coat makes them matte have higher quantity of CHCs in comparison to gynes. In fact, the extra CHC layer covers the cuticle and masks the original brightness of the mated queen, whereas sterile workers are always shiny [24]. It has been suggested that such CHC layer can give extra protection to mated queens against environmental constraints [25]. Furthermore, differences in the CHC profile in mated-fertile queens in terms of the total profile or in relation to a particular or some compounds of the blend may provide odor cues to nestmate workers acting as a fertility signal [26]–[30].

Direct evidences about the mechanism of CHC origin consider the oenocytes [31] as the site of CHC production in insects [20], [32]–[34]. Through *de novo* synthesis assays using dissociated cells Fan et al. [35] conclusively showed the biosynthesis of CHCs by oenocytes in the German cockroach, *Blattella germanica*. This found excluded the hypothesis of the epidermal glands as a possible site of the CHC production [33]. The transport of the CHCs to the target organs as cuticle surface and ovaries is mediated by the lipophorin, a hemolymph lipoprotein [20]. In this way, it is implicit that in social insects the CHCs implicated in the nestmate recognition system as well as in the fertility cues are produced in the oenocytes [e.g. 36].

All the same, other CHC sources were suggested in ants, as the subepithelial glands [37] and fat body [38]. Also, some works mention the glandular status of the epidermis, so named glandular epithelium that could be considered as another potential source of fertility signals in ants. In the Ponerinae ant *Pachycondyla analis* ( = *Megaponera foetens*), the typical glandular epithelium is exclusive to the ergatoid queen whose cuticle is penetrated by several ducts where large setae are innervated. These setae may also serve as dispensers of the secretions in addition to their mechanoreceptor function. Because the queen is highly attractive to workers and workers never show such glands, the epidermal gland might be the source of the queen's signal [39]. Likewise, a recent study states the glandular role of the epidermis in the abdominal sternites of *Dinoponera lucida* (Ponerinae) and among the functions hypothesized for this gland in such species comprise the synthesis, transfer and/or the transformation of the CHCs after production by oenocytes [40]. This last function could explain, for example, the differences on CHC profiles found between reproductive dominant and non-reproductive subordinate individuals [40].

In light of this assumption we have investigated in the present study the relation of the epidermis and the female reproductive status in *E. tuberculatum*, i.e., if the ovarian development linked to the extra CHC production (wax coat) [24] corresponds to changes on the glandular aspect of the epidermis. We used techniques of both light and transmission electron microscopy.

## Methods

### Ant collection and samples

*Ectatomma tuberculatum* colonies were obtained from the Centro de Pesquisa do Cacau (CEPEC/CEPLAC) in Itabuna, State of Bahia, Brazil. Throughout the more than 10 years working with this ant in the laboratory it was possible to distinguish mated-fertile queens from gynes (virgin queens) on the basis of their cuticle appearance: mated queens are matte whereas virgin ones are shiny [24].

In this study we distinguished different types of females: queens [i.e., mated, highly fertile females (>20 yolky oocytes); matte; n = 8], gynes [i.e., unmated queens, slightly or non-active ovaries (<8 yolky oocytes); shiny; n = 8], and workers (i.e., without functional spermathecae or active ovaries; shiny; n = 5). Surprisingly, four unmated queens from three queenless colonies, i.e., colonies without mated females, showed an intermediate wax coat: they presented a smaller amount of this compared to mated-fertile queens but such wax coat was sufficient to render them less shiny that typical gynes. Such atypical unmated females had ovaries highly active similar to those of mated queens. As they differed from typical shiny gynes in relation to visual appearance and ovarian status they were also studied.

To comparison we have also inspected mated and virgin queens of two closed related species: *Ectatomma brunneum* and *Ectatomma vizottoi*. The samples were collected respectively in Itabuna, State of Bahia, and Dourados, State of Mato Grosso do Sul, Brazil (all studied specimens were identified by Jacques H.C. Delabie and vouchers are deposed in the Collection of Laboratório de Mirmecologia). We analyzed a total of five mated and 14 virgin queens of *E. brunneum* and *E. vizottoi*. In these two species, only mated queens had developed ovaries. Contrarily to *E. tuberculatum*, no difference in the cuticle appearance was found in the both species in regard to the wax coat among queens.

### Histology and Ultrastructure

Morphological analyses were performed to study if epidermis differs among females in regard to their reproductive status and their cuticle aspect (matte or shiny). Dissections were done in 125 mM NaCl solution under a stereomicroscope allowing the separation of the pieces (i.e., head, thorax, abdominal tergites, and sternites) and the confirmation of the ovarian and mating status (i.e., full or empty spermathecae) of females.

The pieces of each sample were removed, transferred to 2.5% glutaraldehyde in sodium cacodylate buffer 0.1 M for transmission electronic microscopy analyses or to Zamboni fixative solution [41] for light microscopy. Thus, all pieces head, thorax, abdominal tergites, and sternites were studied in light and electron microscopes.

For histological studies, the samples were dehydrated in a graded ethanol series, and embedded in historesin (Leica). A set of sections 4–5 µm thickness of all pieces of each female were stained with hematoxyline and eosin or toluidine blue-borax. Other sections were submitted to Nile blue, mercury–bromophenol blue and PAS histochemical tests [42] to detect lipids, proteins and neutral carbohydrates, respectively. Samples were analyzed in light microscope.

For ultrastructural studies, some pieces from different female types were post-fixed for 2 h in 1% osmium tetroxide in sodium cacodylate buffer and dehydrated in a graded acetone series. The samples were then embedded in one to the following resin: Epon Araldite or Spurr. Ultra-thin sections were stained with uranyl acetate and lead citrate [43] and they were analyzed with a transmission electron microscope Zeiss EM 109.

## Results

**Figure 1:**
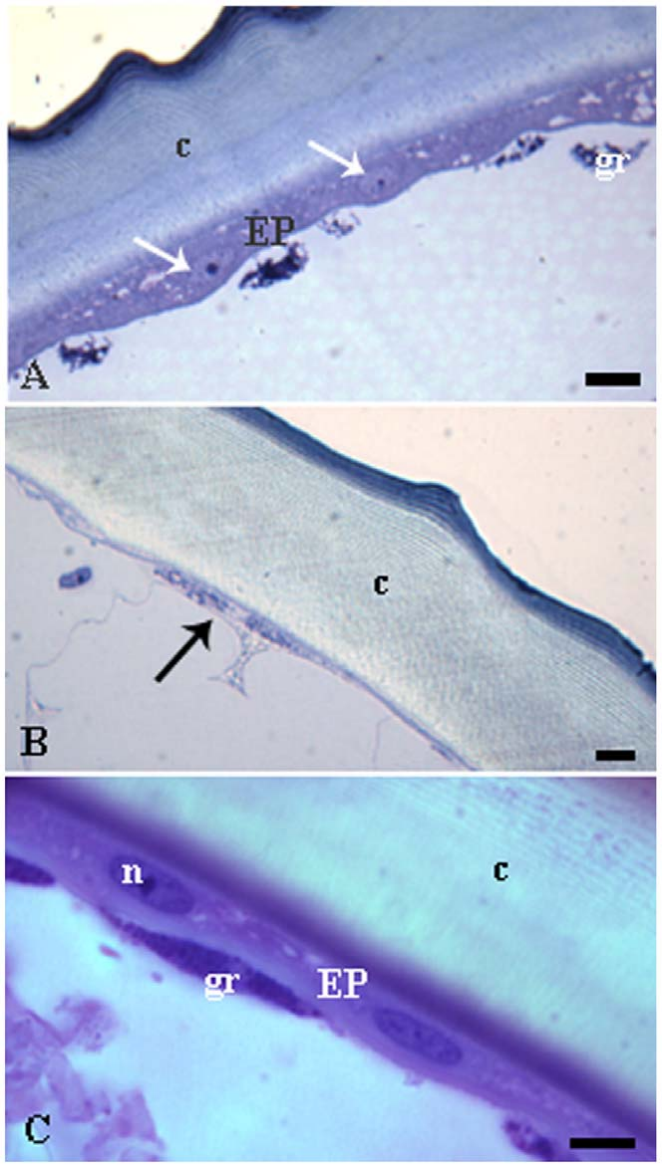
Histological sections of the tergite I of *Ectatomma tuberculatum*. A) Queen showing a cubic glandular epithelium (EP) with well-developed round nuclei (arrows). –B) Gyne showing a thin epidermis with flattened cells and their nuclei (arrows). C) Unmated fertile queen showing an epidermis with elongated cells (EP) and elliptical nucleus (n). c–cuticle, gr–granulocytes associated to epidermis. Bars = 10 µm.

The epidermis of all mated-fertile queens of *E. tuberculatum* inspected clearly differed from those found to typical shiny gynes and workers, and such difference was observed for all samples. The epidermis of mated-fertile queens is characteristically a thick single layer formed by cubic cells (c.a. 15 µm), which corresponds to a thin layer of flattened cells (c.a. 5 µm) in typical shiny gynes and workers (Figures 1A, 1B). The thick layer observed in queen's epidermis was found in all body segments checked, i.e., head, thorax, and all abdominal tergites and sternites. In the four unmated but highly fertile queens the epidermis showed intermediated (c.a. 8 µm) between those characteristic to mated-fertile queen and typical shiny gynes (Figure 1C). In females other than fertile queens, the epidermis was always thin, characterized by flattened cells, regardless of the body region analyzed. The same flattened epidermis characterized the *E. brunneum* and *E. vizottoi* queens independently of their mating or ovarian status.

**Figure 2:**
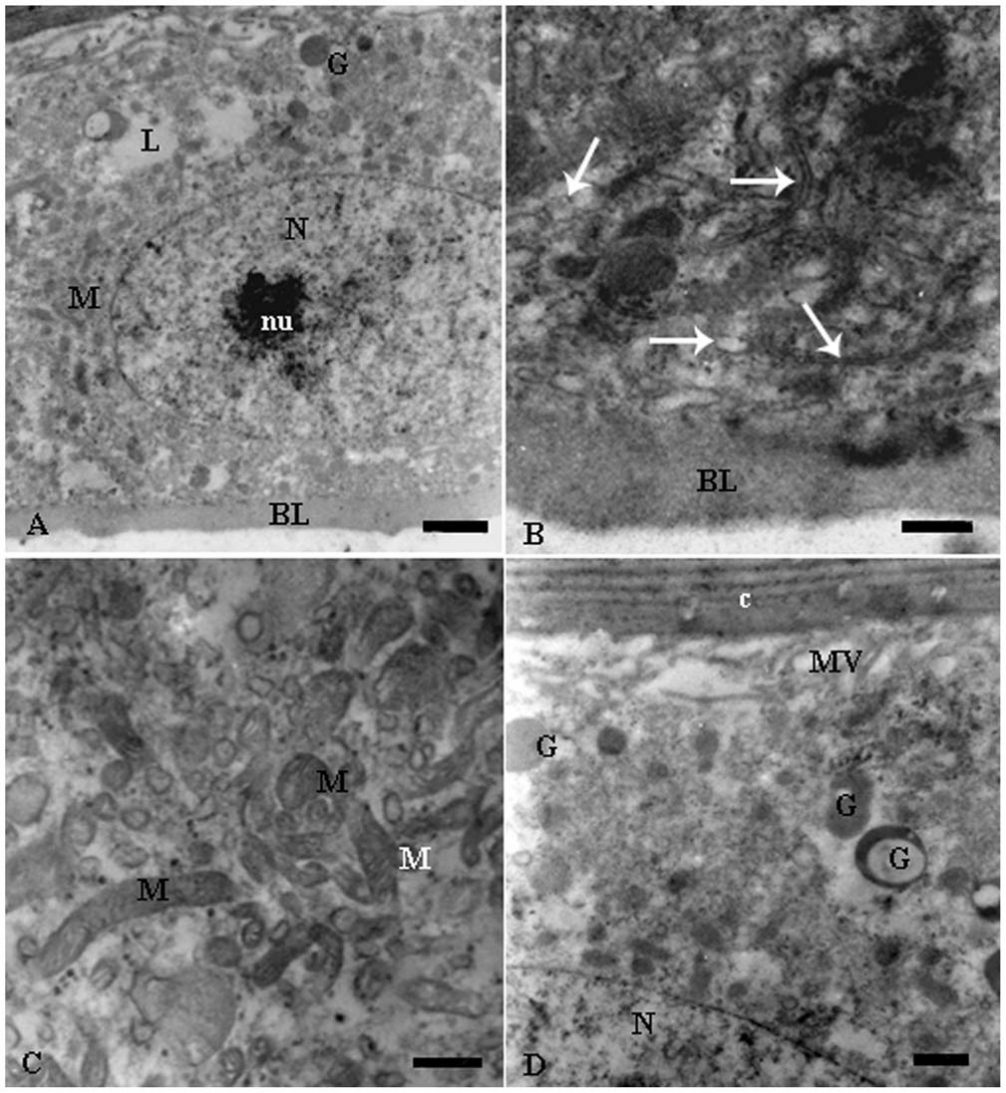
Electron micrographs of queen epidermal gland cells of of *Ectatomma tuberculatum*. A) General view of a gland cell showing the nucleus with decondensed chromatin (N) and well developed nucleolus (nu) and cytoplasm with lipid droplets (L), mitochondria (M) and secretory granules (G) in the epidermis of tergite III. Bar = 1 µm. B) Middle basal region of a gland cell showing smooth endoplasmic reticulum (arrows) in the epidermis of sternite III. Bar = 0.5 µm. C) Detailed view of the middle cell region with many mitochondria (M) in the epidermis of sternite IV. Bar = 1 µm. –D) Apex of a gland cell showing granules (G) with different electron densities and some microvilli (MV) in the epidermis of tergite III. Bar = 0.5 µm. BL, basal lamina; c, body cuticle.

**Figure 3:**
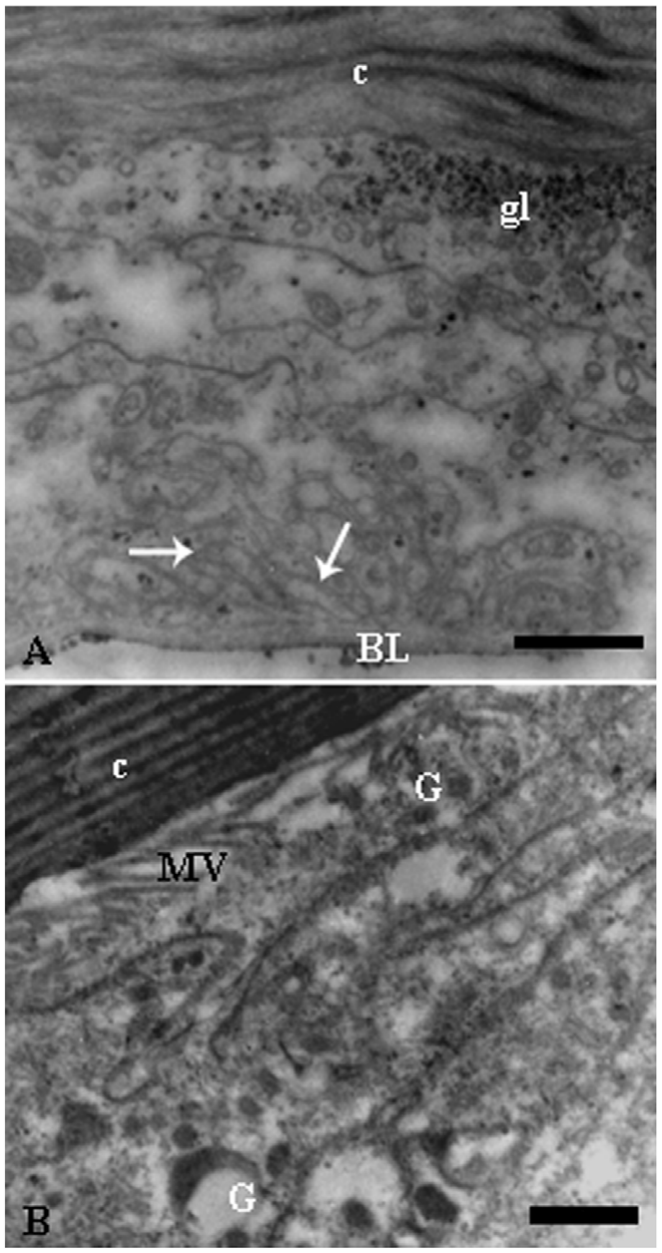
Electron micrographs of queen epidermal gland cells of of *Ectatomma tuberculatum*. A) General view of epidermal gland cell showing some glycogen granules (gl) and well developed basal plasma membrane infoldings (arrows) in the epidermis of tergite IV. Bar = 5 µm. –B) Apex of a gland cell showing a subcuticular space with cell microvilli (MV). Notice granules (G) with different sizes and electron densities in the epidermis of sternite I. Bar = 1 µm. BL, basal lamina; c, body cuticle.

The ultrastructure showed that the epidermis of fertile queens had round nuclei with decondensed chromatin (Figure 2A) and the cell cytoplasm was found to be particularly rich in smooth endoplasmic reticulum, elongated mitochondria, lipid droplets and granules with different electron-densities (Figures 2B, 2C, 2D). The basal cell region was characterized by short plasma membrane infoldings while the cell apex showed small microvilli (Figures 3A, 3B). No excretory canal was observed. The characteristics above support the gland status of the epidermis, so named glandular epithelium, in fertile queens of *E. tuberculatum*.

**Figure 4:**
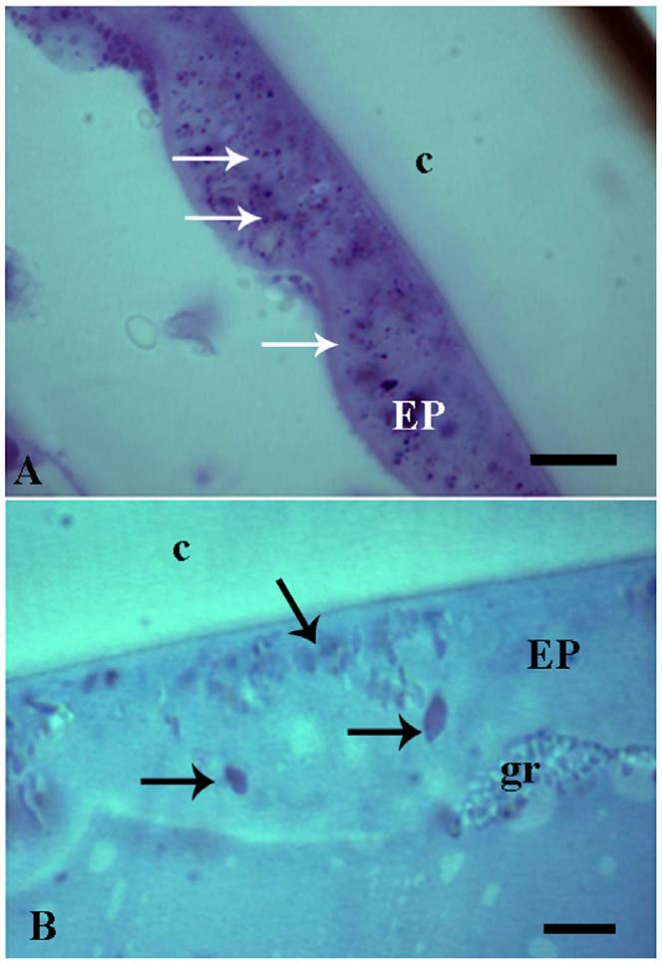
Histochemical tests of queen epidermis of *Ectatomma tuberculatum*. A) PAS test showing strong positive reaction for neutral polysaccharides (arrows) in the epidermis of tergite III. Bar = 10 µm. B) Nile blue test showing positive reaction for neutral lipids (arrows) in the epidermis of tergite II. Bar = 5 µm. c, body cuticle; EP, epidermis; gr, granulocyte associated with epidermis.

The epidermis of fertile queens showed weakly reaction to mercury-bromophenol blue indicating that they are not producing significant amounts of proteins. However, several granules stained with the PAS and Nile blue tests suggesting occurrence of neutral polysaccharides and lipids, respectively (Figures 4A, 4B). Due to their thin epidermis the histochemical analysis were not possible in gynes and workers.

**Figure 5:**
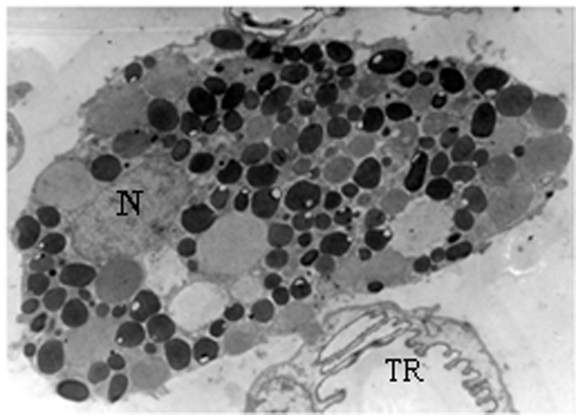
Electron micrograph of a granulocyte associated with queen epidermis of the sternite II of *Ectatomma tuberculatum*. Notice the large amount of cytoplasm granules and the nucleus (N). TR–Trachea. Bar = 5 µm.

We found large amounts of granulocytes adhered to the epithelium, in all female types (Figures 1A, 4B). They had large cytoplasm with many granules and lipid droplets, well developed rough endoplasmic reticulum, Golgi region and many membrane-bounded electron-dense granules (Figure 5). The nucleus presented chromatin uniformly distributed. Granulocytes were also positive for PAS and mercury-bromophenol blue test, showing the presence of carbohydrates and proteins, respectively, in their granules.

## Discussion

The results of our morphological and ultrastructural analyses suggest that the epidermis in queens of *E. tuberculatum* is an exocrine gland of type I according the classification of Noirot and Quennedey [31], representing therefore a glandular epithelium. Only highly fertile queens of *E. tuberculatum*, all producing extra wax coat, presented it. The glandular epithelium in this species could be responsible for the secretion of the compounds characterized as lipid because they contained developed smooth endoplasmic reticulum, in which lipids' synthesis occurs, in addition to lipid droplets found in the cytoplasm that was confirmed by the histochemical tests (Nile blue). Besides, the presence of abundant mitochondria and basal plasma membrane infoldings corroborates the glandular role of the epidermis with an active metabolism and secretion. The absence of glandular duct suggested that the secretion is directly discharged at the cuticle surface by means of cuticular pores (intrinsic pores of the cuticle resulting from its deposition during molt). Also, the lack of the glandular epithelium and the extra wax coat in all queens and gynes of *E. brunneum* and *E. vizottoi* studied reinforce the link between them.

The granulocytes or granular cells found represent a type of hemocytes. Granules seem to be synthesized by the Golgi region and the final stage of their maturation is the electron-dense body [3]. The granulocytes are reported to be phagocytic and may be also involved in nodulation and encapsulation of bacteria, parasitoids and nematodes as well as in melanization process [44]–[46]. The large amount of granulocytes found adhered in *E. tuberculatum* epithelium may be related to those functions, although, more analyses need to be done.

In ants, the glandular epithelium can be found on different body parts, as for example, antennae [47], coxae [48], mandible [49], and infrabuccal cavity [50] though the exact functions of their respective secretions are unknown. Likely we found in highly fertile queens of *E. tuberculatum* a well-developed epidermis like those that have been described in *M. foetens* (Hölldobler et al., 1994) and *D. lucida* [40] where a link with the queen signaling mechanism was suggested. However, it has been largely demonstrated a strong association between CHC profiles and the reproductive status of females in ants [e.g. 27], [28], [51]–[53] as well as in other social insects. Direct evidence of this was elegantly showed in the ponerine ant *Pachycondyla inversa* in a study including electrophysiological experiments [54]. The reproductive signal therefore appears to be a truly reliable signal (i.e., an “honest signal”) because it allows workers to reliably assess to queen fertility [55]. The same correlation was found for *E. tuberculatum* queens, where differences in CHC of mated-fertile queens and infertile gynes were mainly in the relative proportions of nonacosane and heptacosane; also, mated-fertile queens have greater amounts of total CHC covering the cuticular surface [24].

In ants, oenocytes play an important role in protein and lipid synthesis and storage [56], [57]. As these cells are recognized as the unique gland responsible for the CHC biosynthesis [35], what would be thus the function of the glandular epithelium in *E. tuberculatum*? Why do only fertile queens with wax coat show such gland? The answer may lie in the implication of the glandular epithelium in the non-CHCs lipid synthesis. In wax-producing bees it is well known that the glandular epithelium, similar to that found here, is the site of wax biosynthesis [13], [58]–[60]. Such wax that is used in nest building corresponds to the main source of recognition cues in *Apis mellifera* [61], [62]. Wax could therefore be involved in recognition mechanisms by constituting a persistent scented source as proposed by the “scented candle” model [61], [63] for nestmate recognition system. In paper wasps, nest material can trap environmental and genetic resultant substances and continuously release these chemical cues, providing a constant recognition label [63]. This model makes analogy to the perfumed candle that releases a scent slowly [61]. Based on this, some lipids like wax esters that could not be identified by earlier analyses in *E. tuberculatum* [24] might be released by the glandular epithelium and thus adsorb and impregnate hydrocarbons produced by oenocytes and as result queens could more efficiently signal their presence. In fact, oenocytes and fat body were present in larger quantity in fertile *E. tuberculatum* queens compared to gynes and workers (data not shown). In *E. tuberculatum* queens a corresponding system might provide a consistent source of queen's odor that is particularly adaptative in this species where budding and queen displacement can take place [64], [65].

Alternatively, the glandular epithelium may be involved in the synthesis of compounds with another chemical nature and perhaps no detected by our histochemical analysis, which could be involved in the queen recognition mechanism, like suggested to *P. analis* and *D. lucida* [39], [40]. Indeed, other non-CHC cuticular substances, such as proteins, might also be used as chemical recognition cues as it was demonstrated in the paper wasp, *Polistes dominulus*, in the shelter (hibernacula) marking phenomenon [66]. Recently it was also showed in this same species that the foundresses can be distinguished from the workers on the basis of the pattern of cuticular polar peptide compounds, revealing thus the implication of substances other than CHCs as reliable cue to signal the foundresses, although no link with fertility status was found [67]. From this perspective, the chemicals involved in recognition cues in social insects are not exclusive to CHCs and actually the glandular epithelium in *E. tuberculatum* queens could represent a site of synthesis of another queen or fertility signal that could act concomitantly with the CHCs or reinforce the action of them. In fact, in the Myrmeciinae ant *Myrmecia gulosa* nonhydrocarbon polar lipids were suggested to intensify the activity of the CHCs in the queen recognition [27]. Moreover, the unexpected fact that non-inseminated fertile gynes of *E. tuberculatum* showed extra wax coat and the epidermis thicker than that found to typical shiny gynes corroborates with the assumption that queen signaling reflects fertility (ovarian activation) and not mating status [68], [69].

Considering the multifunctional nature of the CHCs we cannot rule out the hypothesis that the glandular epithelium represents a site of CHC production or regulation in *E. tuberculatum* [40]. CHCs implicated in the waterproofing function, in the chemical communication (pheromone function) or in the synthesis of other pheromones (pheromone precursors) could be biosynthesized or regulated in different sites. Thus, further investigations should examine the link between the glandular epithelium and the whole cuticular compounds, in order to better understand queen signaling mechanisms and social insect's chemical communication in general.
